# On-site Dining in Tokyo During the COVID-19 Pandemic: Time Series Analysis Using Mobile Phone Location Data

**DOI:** 10.2196/27342

**Published:** 2021-05-11

**Authors:** Miharu Nakanishi, Ryosuke Shibasaki, Syudo Yamasaki, Satoshi Miyazawa, Satoshi Usami, Hiroshi Nishiura, Atsushi Nishida

**Affiliations:** 1 Research Center for Social Science & Medicine Tokyo Metopolitan Institute of Medical Science Setagaya-ku, Tokyo Japan; 2 Department of Psychiatric Nursing Tohoku University Graduate School of Medicine Sendai-shi, Miyagi Japan; 3 Division of Environmental Studies Department of Socio-Cultural Environmental Studies The University of Tokyo Kashiwa-shi,Chiba Japan; 4 Technology Department LocationMind Inc Chiyoda-ku, Tokyo Japan; 5 Center for Research and Development of Higher Education The University of Tokyo Bunkyo-ku, Tokyo Japan; 6 School of Public Health Kyoto University Kyoto-shi, Kyoto Japan; 7 Tokyo Center for Infectious Disease Control and Prevention Shinjuku-ku, Tokyo Japan

**Keywords:** COVID-19, mobility data, on-site dining, public health and social measures, public health, mobile phone, mobility, protection, time series, location, infectious disease, transmission

## Abstract

**Background:**

During the second wave of COVID-19 in August 2020, the Tokyo Metropolitan Government implemented public health and social measures to reduce on-site dining. Assessing the associations between human behavior, infection, and social measures is essential to understand achievable reductions in cases and identify the factors driving changes in social dynamics.

**Objective:**

The aim of this study was to investigate the association between nighttime population volumes, the COVID-19 epidemic, and the implementation of public health and social measures in Tokyo.

**Methods:**

We used mobile phone location data to estimate populations between 10 PM and midnight in seven Tokyo metropolitan areas. Mobile phone trajectories were used to distinguish and extract on-site dining from stay-at-work and stay-at-home behaviors. Numbers of new cases and symptom onsets were obtained. Weekly mobility and infection data from March 1 to November 14, 2020, were analyzed using a vector autoregression model.

**Results:**

An increase in the number of symptom onsets was observed 1 week after the nighttime population volume increased (coefficient=0.60, 95% CI 0.28 to 0.92). The effective reproduction number significantly increased 3 weeks after the nighttime population volume increased (coefficient=1.30, 95% CI 0.72 to 1.89). The nighttime population volume increased significantly following reports of decreasing numbers of confirmed cases (coefficient=–0.44, 95% CI –0.73 to –0.15). Implementation of social measures to restaurants and bars was not significantly associated with nighttime population volume (coefficient=0.004, 95% CI –0.07 to 0.08).

**Conclusions:**

The nighttime population started to increase after decreasing incidence of COVID-19 was announced. Considering time lags between infection and behavior changes, social measures should be planned in advance of the surge of an epidemic, sufficiently informed by mobility data.

## Introduction

As specific treatment and prevention measures have yet to be established, the COVID-19 global pandemic, caused by SARS-CoV-2, has required nations to implement public health and social measures, strategically focusing on mobility restrictions. SARS-CoV-2 is transmitted primarily by respiratory droplets via close face-to-face contact [1]. As the infection can be spread by asymptomatic and presymptomatic carriers, public health and social measures are warranted that target physical distancing and minimizing verbal interactions [2]. Many countries implemented national lockdowns in early 2020 that have had significant effects on reducing transmission [3]. Early and intensive interventions with lockdown periods are effective in reducing clinical cases and preventing an excess of health care demand compared with the supply [4]. However, once the lockdown measures were relaxed, second waves of COVID-19 gradually started, and large parts of Europe were forced to decide whether to implement second lockdowns [5]. National lockdowns and human mobility restrictions can potentially have immense adverse economic effects [6]. Governments worldwide now face the common challenge of easing lockdowns and restrictions while balancing out damages in various sectors, including health, social, and economic aspects [7]. Less restrictive public health and social measures to suppress the COVID-19 epidemic are called for, and such early interventions that are focused on reducing specific high risk behaviors are being explored.

Dining at restaurants, bars, and nightclubs involves removal of face masks and talking with others; thus, this activity is associated with elevated risk of COVID-19 transmission [8]. At a national level, in September 2020, restaurants, bars, and pubs in England were required to close at 10 PM [9]. Local measures to close bars and restaurants were also implemented in France from September 2020 [10]. In Japan, the national government’s response to the second wave during July-August 2020 appeared to be slow, as the Go To Travel campaign that offered discounts on hotel charges and local coupons to encourage consumption was launched on July 22 [11]. The Tokyo Metropolitan Government asked restaurants and bars to close at 10 PM from August 3 to September 15, 2020 [12]. Tokyo metropolitan areas have not only the highest cumulative incidence of COVID-19 but also the highest population density [13], which is now recognized as the factor that determines the secondary transmission of COVID-19, and this area faced a second wave of COVID-19 starting in July 2020 [12]. To date, it has remained unclear how successfully public health and social measures can be implemented to reduce specific behaviors with high risk of infection (ie, on-site dining). Assessing the associations between human behavior, infection, and public health and social measures is therefore essential to understand achievable reductions and identify the factors driving the changes in social dynamics. The findings of such an assessment can have global implications for public health and social measures to suppress COVID-19.

Mobile phone location data can be used to monitor changes in human behavior across different locations in a country. These data indicate whether people are staying in the same location or moving around. The effects of public health and social measures to suppress COVID-19 transmission range from reduction of travel to distant locations to increased fractions of people staying at home, as assessed by mobile phone location data [14,15]. However, there is limited evidence regarding less restrictive COVID-19 countermeasures and social dynamics related to on-site dining. In this study, we used mobile phone trajectories to estimate the nighttime population volumes of people who stayed near restaurants and bars as a measure of on-site dining behavior. We investigated the association between nighttime population volumes, the COVID-19 epidemic, and the implementation of public health and social measures in Tokyo.

## Methods

### Timeline of the COVID-19 Pandemic in Tokyo

Infection data were collected during a 34-week period from March 1 to November 14, 2020. The study period was determined to observe the initial confirmed case reports in Tokyo, followed by the implementation of self-restraint–based contact reduction across all areas of Tokyo and social measures in restaurants and bars by the Tokyo Metropolitan Government. The number of new COVID-19 case reports and symptom onsets per day was made publicly available at websites by the Tokyo Metropolitan Government [16]. The number of symptom onsets was sequentially added over 3-week periods of prompt reports, as there were several time lags between symptom onset, testing, diagnosis, and inclusion in the daily report of new cases. We thus obtained data on the number of symptom onsets as of December 5, 2020. In this study, the weekly numbers of symptom onsets and confirmed case reports were used for analysis.

The effective reproduction number (*R_t_*), the average number of secondary cases generated by a single primary case, was estimated as a function of the estimated calendar date of infection. To achieve this, the date of infection was statistically inferred using confirmed cases that were divided into two groups: cases with (1) known date of illness onset and (2) known date of confirmation and without known date of illness onset. For the latter group, we back-projected the suggested date of illness onset nonparametrically, and then we summed these cases with group (1). Subsequently, we implemented nonparametric back-projection of the date of infection using the incubation period distribution with a mean of 5.2 days. Using the assumed median generation time of 4.6 days [17], the renewal equation was employed to estimate everyday *R_t_*. The Bayesian Markov chain Monte Carlo method was implemented using rstan, version 2.19.3; the estimation code is available in the GitHub repository [18]. To control for local heterogeneities that are exhibited as daily fluctuations, we smoothed the time series by computing the 7-day rolling averages, and we adopted the number on the fourth day of each week for analysis.

### Mobile Phone Location Data

Population volumes at 10 PM to midnight in seven Tokyo metropolitan areas were estimated using location data from smartphones created by LocationMind xPop. LocationMind xPop uses aggregated people flow data originally collected by NTT Docomo, Inc, through their application service “Docomo Map Navi” using only cell phone location data collected with user consent to the auto GPS function of the service, and the data are then statistically processed by NTT Docomo in their entirety before being provided to LocationMind Inc [19]. The original location data are GPS data (latitude and longitude) sent at a frequency of every 5 minutes at the shortest interval, and these data do not include information that specifies individuals [20]. NTT Docomo is the largest mobile phone operator in Japan, and it accounts for approximately 44% of total mobile phone subscribers [21]. The population per hour was estimated using the location data and calibration with the national census.

The users in this study agreed to provide their location information. The data are anonymized so that individuals cannot be specified, and personal information such as gender, age, and occupation are unknown. The population in this study represents the number of mobile devices that appear in a 500 m^2^ grid at a specific time. Mobile phone trajectories were used to distinguish and extract on-site dining behavior from stay-at-work and stay-at-home behaviors. A stay point was assumed at which the GPS data of a device are concentrated within the area with positional errors for approximately 15 minutes or longer. A user’s place of work and home were estimated using the information on the stay points, including period of time and length of stay per user. Stay points that were spatially distinct from the estimated place of work and home were accounted for with stay episodes other than stay-at-work and stay-at-home. The following seven Tokyo metropolitan areas were selected to represent districts with restaurants and bars: Kabuki-cho (mesh code 533945361), Ginza-Corridor-gai (mesh code 533946002), Shibuya-Center-gai (mesh code 533935952), Ueno-Nakamachi-dori (mesh code 533946512), Shinjuku-Ni-chome (mesh code 533945264), Ikebukuro (mesh code 533945764), and Roppongi (mesh code 533935984). The selection was based on designated areas in Tokyo for monitoring of people flow data by the Cabinet Office [22].

### Nighttime Population Volume

Because using a single data source at a specific time may not represent the population distribution in the whole period, the average population concentration was used to estimate the nighttime population volumes in seven Tokyo metropolitan areas between 10 PM and midnight. A scaling factor was calculated using the estimated place of home for each user in combination with the national census, which gave a value of the total population represented by the user. The number of people staying in a certain area per hour was calculated by summing the scaling factors of the users. The estimated number of people per hour was calculated on a weekly basis to meet a sufficient sample size, as areas that contain few persons should be removed to prevent the identification of individuals based on their location.

### Public Health and Social Measures

During the first wave of COVID-19, self-restraint–based contact reduction was implemented for the entire population of Tokyo for a 7-week period from April 7 to May 24, 2020. First-wave countermeasures included declaration of a state of emergency, call for self-restraint in travel and going out, restrictions of the maximum capacity of events and gatherings, and restrictions of use of facilities where clusters were occurring and the “3Cs” (closed space, crowded space, and closed contact setting) were observed [23]. Restaurants and bars were asked to cooperate by shutting down their services at 8 PM during this period.

During the second wave, social measures for restaurants and bars in Tokyo were in place for a 6-week period from August 3 to September 15, 2020. Restaurants and bars serving alcohol were asked to shorten their operating hours to 10 PM for this 6-week period.

Neither the first nor the second wave interventions were legally binding; for example, owners of restaurants and bars were asked to cooperate with the shutdown of services, and instead, the owner received a subsidy from Tokyo. In the analysis, the implementation of public health and social measures was presented as a dichotomous variable (implemented=1, absent=0).

### Statistical Analysis

The initial and peak weeks were identified by visual inspection of the observed time series in the night-time population, symptom onsets, confirmed case reports, and *R_t_*.

A vector autoregression (VAR) model was employed to predict endogenous outcomes (ie, nighttime population volume, symptom onsets, confirmed case reports, *R_t_*) while investigating how they are influenced by each other in terms of Granger causality [24]. VAR is a system of equations with outcomes that depend on other outcome variables. Suppose we have a vector of time series data Wt, Xt, Yt, and Zt (four endogenous variables); then, the VAR model with one exogenous variable and p lags can be expressed as below:

Wt = ν1 + ρ1Ct + α11Wt-1 + … + α1pWt-p + β11Xt-1 + … + β1pXt-p + γ11Yt-1 + … + γ1pYt-p + δ11Zt-1 + … + δ1pZt-p + υ

Xt = ν2 + ρ2Ct + α21Wt-1 + … + α2pWt-p + β21Xt-1 + … + β2pXt-p + γ21Yt-1 + … + γ2pYt-p + δ21Zt-1 + … + δ2pZt-p + υ2

Yt = ν3 + ρ3Ct + α31Wt-1 + … + α3pWt-p + β31Xt-1 + … + β3pXt-p + γ31Yt-1 + … + γ3pYt-p + δ31Zt-1 + … + δ3pZt-p + υ3

Zt = ν4 + ρ4Ct + α41Wt-1 + … + α4pWt-p + β41Xt-1 + … + β4pXt-p + γ41Yt-1 + … + γ4pYt-p + δ41Zt-1 + … + δ4pZt-p + υ4

where ν is the intercept, ρ is the coefficient of exogenous variable C, α is the coefficient of the lagged terms of W, β is the coefficient of the lagged terms of X, γ is the coefficient of the lagged terms of Y, δ is the coefficient of the lagged terms of Z, and υ is assumed to be white noise. In the W equation, β, γ, and δ infer how W is influenced by other outcomes (X, Y, and Z) in terms of Grander causality.

The number of lags can be statistically evaluated by information criteria such as the Akaike information criterion (AIC). To measure the effect of restrictions on growth in the nighttime population and infections, the first difference in the natural log was used for a respective time series to stabilize the variances with removing trends, as was applied in a published study [25]. Regarding *R_t_*, the first difference was used for analysis. First-wave countermeasures and social measures implemented by restaurants and bars were included in the VAR model as exogenous covariates. Of interest to our research was the effect of the implementation of restrictions on restaurants and bars on the nighttime population volume and the lagged coefficient of the nighttime population volume in symptom onsets and *R_t_*. Granger causality tests were performed for each equation to assess whether any pairs among nighttime population volume, symptom onsets, confirmed case reports, and *R_t_* had a causal relationship.

A sensitivity analysis was conducted using the vector autoregression model in which the symptom onsets and confirmed case reports were excluded from the endogenous variables.

All statistical analyses were performed using Stata, version 16.1 (StataCorp LLC). The significance level in the two-tailed tests was set to .05.

## Results

**Figure 1:**
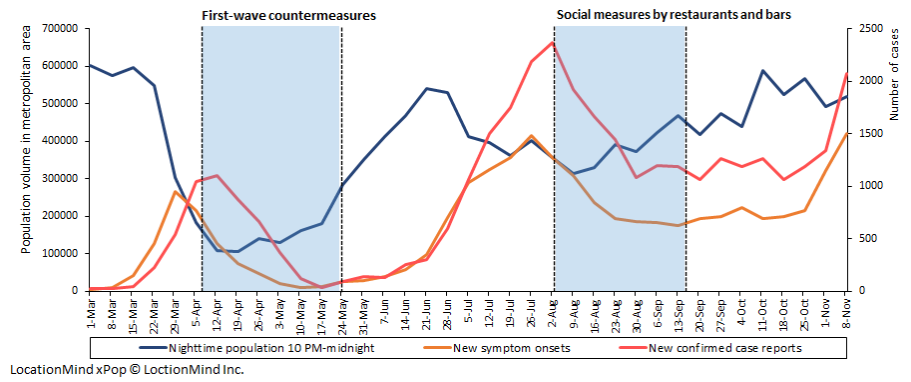
Nighttime population volumes in Tokyo metropolitan areas, number of new symptom onsets, and confirmed case reports of COVID-19 per week. LocationMind xPop © Location Mind Inc.

**Figure 2:**
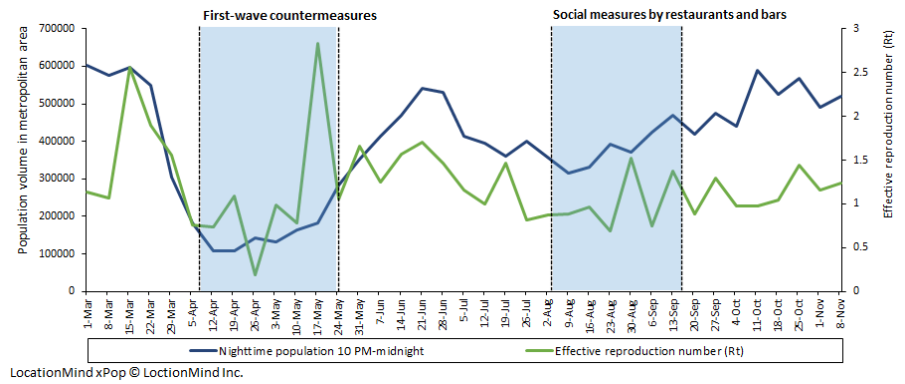
Nighttime population volumes in Tokyo metropolitan areas and the effective reproduction number of COVID-19 per week. The blue line presents the sum of the estimated numbers of people in the seven districts between 10 PM and midnight. The green line presents the effective reproduction number based on the number of confirmed cases in Tokyo. LocationMind xPop © Location Mind Inc.

The sum of the nighttime population volumes of the seven districts appeared to exhibit a decreasing trend before the implementation of first-wave countermeasures. During the self-restraint period, this sum started to increase, and it peaked around late June 2020. During the period of restrictions on restaurants and bars, the nighttime population volume consistently increased over time (Figure 1). The estimated volume of the nighttime population per area showed similar time trends from one place to the other (Multimedia Appendix 1). The first peak of confirmed case reports was observed in late March, and the second peak was around late July 2020 (Figure 1). Overall, the peak of confirmed case reports followed symptom onsets with a 3-week lag. The total number of symptom onsets during the study period was 22,902, accounting for 66% of confirmed case reports (n=34,467). The pattern of *R_t_* during the study period was similar to that the of the nighttime population volume, with a decreasing trend before the implementation of first-wave countermeasures and social measures in restaurants and bars (Figure 2).

For the lag-length selection of the VAR model, the Schwarz criterion, final prediction error, AIC, and Hannan-Quinn criterion suggest four lags. Consequently, we used four lags in the VAR model for further analysis. The results of the VAR model showed that the increase in *R_t_* was significantly associated with increases in nighttime population volume 3 and 4 weeks prior (Table 1). The increase in symptom onset was significantly associated with increases in the nighttime population volume 1, 3, and 4 weeks prior. Additionally, the increase of the nighttime population volume was significantly associated with a decrease in confirmed case reports 1-4 weeks prior. The implementation of social measures in restaurants and bars was not significantly associated with the nighttime population volume. The implementation of lockdown was significantly associated with an increase in the nighttime population volume.

**Table 1:** Results of vector autoregression analysis on the number of confirmed case reports, symptom onsets, nighttime population volume, and *R_t_* per week.^a^

Variable	Lag	*R_t_* ^b^			Nighttime population volume			Symptom onsets		Confirmed case reports			
		Coefficient (95% CI)	*P* value	Coefficient (95% CI)		*P* value	Coefficient (95% CI)		*P* value		Coefficient (95% CI)	*P* value	
*R_t_*	1	–1.00 (–1.41 to –0.59)^c^	<.001	0.04 (–0.11 to 0.20)		.58	0.30 (0.06 to 0.55)^c^		.02		–0.03 (–0.22 to 0.17)	.80	
	2	–0.17 (–0.76 to 0.42)	.58	–0.16 (–0.39 to 0.06)		.15	0.22 (–0.14 to 0.58)		.22		–0.24 (–0.52 to 0.05)	.10	
	3	0.02 (–0.51 to 0.56)	.93	–0.71 (–0.91 to 0.50)*		<.001	0.12 (–0.20 to 0.44)		.48		–0.14 (–0.40 to 0.11)	.28	
	4	–0.12 (–0.46 to 0.22)	.50	–0.38 (–0.51 to –0.25)^c^		<.001	0.07 (–0.13 to 0.28)		.48		0.08 (–0.09 to 0.24)	.36	
Nighttime population volume	1	0.42 (–0.11 to 0.95)	.12	–0.25 (–0.45 to –0.05)^c^		.01	0.60 (0.28 to 0.92)^c^		<.001		–0.05 (–0.31 to 0.20)	.69	
	2	0.41 (–0.14 to 0.95)	.14	0.29 (0.08 to 0.49)^c^		.006	–0.17 (–0.50 to 0.16)		.30		–0.26 (–0.52 to –0.001)^c^	.05	
	3	1.30 (0.72 to 1.89)^c^	<.001	–0.52 (–0.74 to –0.29)^c^		<.001	0.63 (0.28 to 0.99)^c^		<.001		–0.33 (–0.61 to –0.05)^c^	.02	
	4	0.59 (0.04 to 1.14)^c^	.03	–0.03 (–0.24 to 0.18)		.77	0.59 (0.26 to 0.92)^c^		<.001		0.07 (–0.20 to 0.33)	.62	
Symptom onset	1	–0.57 (–1.25 to 0.11)	.10	0.02 (–0.24 to 0.27)		.90	0.35 (–0.06 to 0.76)		.09		1.40 (1.07 to 1.72)^c^	<.001	
	2	–1.50 (–2.69 to –0.32)^c^	.01	1.05 (0.61 to 1.50)^c^		<.001	–0.47 (–1.19 to 0.25)		.20		0.15 (–0.42 to 0.71)	.62	
	3	1.42 (0.50 to 2.35)^c^	.002	–0.10 (–0.45 to 0.25)		.59	0.12 (–0.44 to 0.68)		.68		–0.35 (–0.79 to 0.09)	.12	
	4	–0.58 (–1.33 to 0.18)	.13	0.38 (0.10 to 0.67)^c^		.008	0.24 (–0.22 to 0.70)		.31		0.45 (0.09 to 0.81)^c^	.02	
Confirmed case reports	1	0.14 (–0.62 to 0.91)	.72	–0.44 (–0.73 to –0.15)^c^		.003	0.06 (–0.40 to 0.53)		.79		–0.37 (–0.74 to –0.003)^c^	.048	
	2	–0.20 (–0.80 to 0.39)	.50	–0.29 (–0.52 to –0.07)^c^		.01	0.25 (–0.11 to 0.61)		.17		0.02 (–0.27 to 0.30)	.91	
	3	–0.22 (–0.70 to 0.26)	.36	–0.65 (–0.84 to –0.47)^c^		<.001	–0.22 (–0.51 to 0.07)		.14		–0.07 (–0.30 to 0.16)	.55	
	4	0.51 (0.18 to 0.84)^c^	.002	–0.31 (–0.44 to –0.19)^c^		<.001	0.07 (–0.12 to 0.27)		.46		–0.18 (–0.34 to –0.02)^c^	.02	
Social measures in restaurants and bars (August 3 to September 15, 2020)	N/A^d^	–0.07 (–0.27 to 0.14)	.52	0.004 (–0.07 to 0.08)		.91	–0.07 (–0.20 to 0.05)		.25		–0.02 (–0.11 to 0.08)	.72	
First-wave countermeasures (April 7 to May 24, 2020)	N/A	0.04 (–0.29 to 0.38)	.82	0.18 (0.05 to 0.30)^c^		.006	–0.03 (–0.24 to 0.17)		.74		–0.05 (–0.21 to 0.11)	.56	

^a^The number of optimal lags was determined by information criteria, including the Akaike information criterion, Hannan–Quinn information criterion, Schiwarz-Bayesian information criteria, and final prediction error.

^b^*R_t_*: effective reproduction number.

^c^Significant at *P*<.05.

^d^N/A: not applicable.

The results of the Granger causality tests indicated the existence of bidirectional causality running from confirmed case reports to the nighttime population, bidirectional causality running from the nighttime population to symptom onsets, and bidirectional causality running from the nighttime population to *R_t_* (Table 2).

**Table 2:** Granger causality tests of pairs between nighttime population, symptom onsets, confirmed case reports, and *R_t_*.

Equation	Excluded	Chi-square (*4*)	*P* value
*R_t_* ^a^	Nighttime population	42.36	<.001
	Symptom onsets	18.12	.001
	Confirmed case reports	13.67	.008
Nighttime population	*R_t_*	67.28	<.001
	Symptom onsets	26.45	<.001
	Confirmed case reports	62.51	<.001
Symptom onsets	*R_t_*	9.42	.051
	Nighttime population	47.71	<.001
	Confirmed case reports	10.36	.04
Confirmed case reports	*R_t_*	14.39	.006
	Nighttime population	12.93	.01
	Symptom onsets	82.23	<.001

^a^*R_t_*: effective reproduction number.

The lagged coefficients of the nighttime population to *R_t_* remained significant (1 week prior, coefficient 1.08, 95% CI 0.28-1.87; 2 weeks prior, coefficient 0.90, 95% CI 0.10-1.70) in the sensitivity analysis, in which symptom onsets and confirmed case reports were excluded from the endogenous variables. The bidirectional causality running from the night-time population to *R_t_* was also significant (χ^2^_4_= 27.00, *P*<.001) in this analysis.

## Discussion

### Principal Results

The increase in the nighttime population in the seven examined districts in Tokyo was followed by increased symptom onsets and increased *R_t_* thereafter. Even during the intervention period, the nighttime population increased significantly following the decrease in confirmed case reports. The implementation of social measures by restaurants and bars was not significantly associated with the nighttime population during the second wave in Tokyo.

The results of our study imply that people adjusted their level of mobility according to the number of confirmed case reports and, thus, their perceived risk of their own infection. They may have reduced on-site dining behavior voluntarily after an increasing number of new confirmed cases was reported and then relaxed their mobility restrictions after a decreasing number of cases was reported. Although people dynamically adjusted their behavior in response to information and policies [26] and recognized that dining inside restaurants should be avoided [27], public attitudes related to behavioral restrictions changed as the COVID-19 epidemic changed. It is notable that there were considerable time lags between behavior changes, symptom onsets, and *R_t_*. A time lag between major mobility reduction and the peak of confirmed cases was also observed in South Korea [28]. There could have been a delay in the voluntary reduction in on-site dining from the time that suppression of the surge of COVID-19 should have begun. This latency was also observed in the reaction to policy measures to restrict mobility in northern Italy [29]. Therefore, policy measures to mitigate COVID-19 should be planned at an earlier stage informed by social behavior dynamics, rather than based on the number of confirmed case reports. Early implementation of strategies is crucial to successful COVID-19 suppression and to avoid national lockdowns [30]. A tracking system of the nighttime population using mobile phone location data would be helpful for policy decision makers to monitor these dynamics in a real-time manner. Space-time dispersions in transmission of COVID-19 have occurred from metropolitan areas towards the countryside [31]. Thus, an automated information system to support strategic policy decision making is of particular importance in metropolitan areas with high population density and mobility, where there is an elevated risk of COVID-19 transmission.

The implementation of first-wave interventions in early 2020 was significantly associated with an increase in the nighttime population volume in Tokyo. Less restrictive measures, requesting that restaurants and bars shorten their operating hours, were not significantly associated with the nighttime population volume. The nighttime population volume started to decrease one month before the implementation of first-wave countermeasures and social measures to restaurants and bars, and it reached the minimum in the early phase of the interventions. People may have reduced on-site dining behavior earlier than the implementation of public health and social measures in response to preceding policy arguments and announcement of the implementations along with the increase in confirmed cases. It should be noted that the decreasing trend appeared to be weaker against the second wave than in March 2020. Although we could not confirm this from our data, many people may have experienced caution fatigue by repeated COVID-19 surges and the highest number of new cases, and they may have become tired of physical distancing. Another possible explanation is that the public health and social measures were suboptimal to suppress the recovery of on-site dining behavior based on the level of the restrictions and the timing of the implementation. However, we could not determine the optimal level of restrictions and length of interventions with regard to COVID-19 mitigation or the economic effects of the interventions. Closure of restaurants and bars may result in decreased consumption expenditure and economic decline. Further investigation is needed to estimate the effect of reductions in the nighttime population volume on the economy and on COVID-19 transmission. This estimation can also inform the government as to how financial compensation should be provided to entities that comply with requests to close at night. In Japan, the national government launched a Go To Eat campaign to encourage people to dine at restaurants starting in October 2020 [32]. This campaign aimed to support the recovery of the industry from economic decline caused by first-wave countermeasures and other behavior restrictions; however, there are concerns that it increased on-site dining and that it resulted in the recent large resurgence of COVID-19 [33]. Strategies to support businesses need to be organized in conjunction with a level of behavior restrictions to prevent COVID-19 resurgence.

### Limitations

This is the first study to relate public health and social measures to prevent COVID-19 transmission to the nighttime population volume in Tokyo. A strength of this study is the estimation of probable on-site dining behavior as opposed to stay-at-work and stay-at-home behavior using mobile phone location data. However, our study has some limitations that must be noted. The suspected mode of transmission in each case was unavailable due to the nature of open data, potentially leading to underestimation of the association between the nighttime population volume and the onset of symptoms. Lags may have been shorter if symptom onset were stratified by transmission via on-site dining. The risk of COVID-19 transmission in on-site dining could also be mitigated over time as restaurants and bars increasingly adopted physical distancing guidelines [34]. The number of reported cases in the first wave might have been suppressed because of limited testing capacity in early 2020. The effectiveness of the measures to reduce on-site dining may vary across regions and countries that have different response levels to policies among the general population. The level of effectiveness would also differ according to the stage of pandemic (first, second, or later), as public health and social measures against later waves can be subject to cumulative caution fatigue against COVID-19. Social behavioral dynamics could also be caused by the media’s reporting on COVID-19 [35]. Future examination on the association between on-site dining and media reporting will be beneficial to provide guidelines to the media that support effective mobility restrictions.

### Conclusions

Regardless of restrictions on restaurants and bars in Tokyo metropolitan areas, people reduced their mobility restrictions after decreases in new confirmed cases were reported. An increase in the nighttime population volume may result in increased symptom onset and *R_t_*. A tracking system of the nighttime population using mobile phone location data would be helpful for policy decision makers to monitor social behavior dynamics and suppress COVID-19 transmission at an earlier stage.

## Appendix

Multimedia Appendix 1 Estimated numbers of the nighttime population in seven Tokyo metropolitan areas between 10 PM and midnight per week from April 7 to May 24, 2020, in Tokyo.

## Abbreviations

AIC: Akaike information criterion
*R_t_*: effective reproduction number
VAR: vector autoregression
