# Supplementary figures and images for: On-site Dining in Tokyo During the COVID-19 Pandemic: Time Series Analysis Using Mobile Phone Location Data
Source: JMIR Mhealth Uhealth. 2021 May 11;9(5):e27342. doi: 10.2196/27342 (PMC8115398; doi:10.2196/27342)

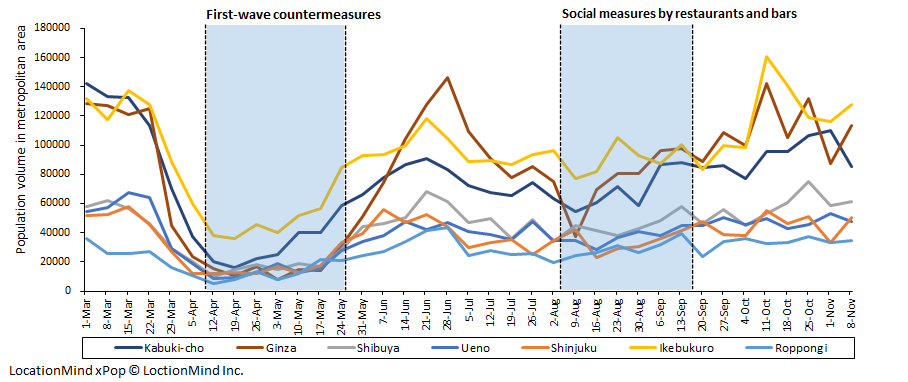

Supplement: Multimedia Appendix 1 [file mhealth_v9i5e27342_app1.png]
